# Navigating dietary small RNAs

**DOI:** 10.1186/s12263-017-0565-3

**Published:** 2017-06-22

**Authors:** Kendal D. Hirschi

**Affiliations:** Children’s Nutrition Research Center Baylor College of Medicine, Houston, USA

## Abstract

When a novel nutritional concept comes along, scientists become enthusiastic and start new explorations. In 2012, the field was enthralled with a study suggesting plant-based nucleic acid “information” acts as a bioactive to regulate animal metabolism.

The field of nutrition can be thought of as a vast ocean that scientists continue to navigate by trial and error (mostly error). Wide-ranging observations have revealed that plant-based diets convey health maintenance and disease prevention benefits. However, the plant ingredients that confer these advantages remain, largely, unidentified. Observational studies have implied that particular bioactive plant food constituents cause the detected benefits; however, 12 randomized controlled trials (RCTs) that tested 52 health promotion or disease prevention claims *all* failed to support the effects implicated by the original studies [5]. A myriad of reasons for these discrepancies could exist; however, the best explanation is that scientists are not properly circumnavigating this deep-sea. It is worth recalling that these errors are not exclusive to nutrition as *Helicobacter pylori* (*H. pylori*), a causal agent of gastric ulcers, was never implicated in epidemiological studies because these reports failed to look for transmissible agents [3].

When a novel nutritional concept comes along scientists become enthusiastic and start new explorations into the seemingly clarified waters. In 2012, the field was enthralled with a study suggesting plant-based nucleic acid “information” acts as a bioactive to regulate animal metabolism [6]. Plant diets contain hundreds of different small RNAs including microRNAs (miRNAs). A microRNA (miRNA) is a tiny (19-24 nucleotide) piece of RNA that attaches to a specific protein-making mRNA thus inhibiting protein production. This initial finding shows that a miRNA in rice survives digestion, circulates through the body, and modulates gene expression. Given that six nucleotides of perfect complementary between the “seed” region of a small RNA and its target are sufficient to promote RNA silencing in mammals, how many of these numerous dietary miRNAs regulate gene expression in consumers? Answering this question *could* alter our understanding of nutrition and trans-kingdom gene regulation and open up new vistas for gene therapy. However, replication and validation of this 2012 observation have not been straightforward causing waning (or lost) enthusiasm among scientists [2]. For the majority of scientists, this field appears to be a shipwreck [4]! Nonetheless, some labs continue to report stirring findings regarding the potential of dietary RNAs [1]. In this issue of Genes and Nutrition, the senior author of the seminal 2012 paper Dr. Chen-Yu Zhang debates the validity of this field with Dr. Ken Witwer, who has published extensively regarding the fields’ limitations. They discuss whether dietary RNA researchers are pioneering an exciting new field or are they simply lost at sea? Dr. Jonathan Snow and Dr. Stephen Chan, who have been unable to validate the bioavailability of dietary miRNAs in their own research, review the literature here and diplomatically propose that the field should be scuttled. A research paper from Kendal Hirschi’s lab, whose group has worked on the bioavailability of plant small RNAs, presents preliminary data suggesting that transgenic miRNAs expressed in plants are not readily bioavailable. Dr. Janos Zempleni, who has worked on the bioavailability of miRNAs from bovine milk, contributes a commentary piece suggesting that the field should pivot slightly and study dietary delivery of different types of nucleic acids, proteins, and lipids. Five years after the initial publication caught the field by storm, these papers can serve as a compass for navigating dietary RNA research.
